# Tools to investigate how interprofessional education activities link to competencies

**DOI:** 10.3402/meo.v20.28627

**Published:** 2015-07-23

**Authors:** Courtney West, Michael Veronin, Karen Landry, Terri Kurz, Bree Watzak, Barbara Quiram, Lori Graham

**Affiliations:** 1 College of Medicine, Texas A&M Health Science Center, Bryan, TX, USA; 2 College of Pharmacy, Texas A&M Health Science Center, Kingsville, TX, USA; 3 College of Nursing, Texas A&M Health Science Center, Bryan, TX, USA; 4 College of Medicine, Texas A&M Health Science Center, Round Rock, TX, USA; 5 College of Pharmacy, Texas A&M Health Science Center, College Station, TX, USA; 6 School of Public Health, Texas A&M Health Science Center, College Station, TX, USA

**Keywords:** interprofessional education, competencies, curriculum development

## Abstract

Integrating interprofessional education (IPE) activities and curricular components in health professions education has been emphasized recently by the inclusion of accreditation standards across disciplines. The Interprofessional Education Collaborative (IPEC) established IPE competencies in 2009, but evaluating how activities link to competencies has not been investigated in depth. The purpose of this project is to investigate how well two IPE activities align with IPEC competencies. To evaluate how our IPE activities met IPEC competencies, we developed a checklist and an observation instrument. A brief description of each is included as well as the outcomes. We analyzed Disaster Day, a simulation exercise that includes participants from Nursing, Medicine, and Pharmacy, and Interprofessional Healthcare Ethics (IPHCE), a course that introduced medical, nursing, and pharmacy students to ethical issues using didactic sessions and case discussions. While both activities appeared to facilitate the development of IPE competencies, Disaster Day aligned more with IPEC competencies than the IPHCE course and appears to be a more comprehensive way of addressing IPEC competencies. However, offering one IPE activity or curricular element is not sufficient. Having several IPE options available, utilizing the tools we developed to map the IPE curriculum and evaluating competency coverage is recommended.

Working effectively with other disciplines is an essential skill for healthcare practitioners. While the importance of interprofessional education (IPE) has been recognized for some time, the move by accreditation bodies in the health professions to incorporate IPE standards (1) has served to propel IPE forward.

In 2009, Core Competencies for Interprofessional Collaborative Practice were developed by the Interprofessional Education Collaborative (IPEC) (2). Institutions have utilized these competencies which are organized into four domains: Values/Ethics for Interprofessional Practice, Roles/Responsibilities, Interprofessional Communication, and Teams and Teamwork, to develop and guide their IPE activities. However, assessing how specific activities align with and address these competencies has not been investigated in depth.

The purpose of this manuscript is to examine the IPEC competencies and analyze how specific IPE activities addressed the competencies. The research question is: How do two IPE activities at the Texas A&M Health Science Center (TAMHSC) align with the competencies delineated by IPEC?

## Methods

Disaster Day (DD) and Interprofessional Healthcare Ethics (IPHCE) were two IPE curricular elements offered at our institution. After receiving Texas A&M University's Institutional Review Board approval, each activity was evaluated for alignment with IPEC competencies. To analyze the activities, two tools, an observation instrument and a checklist, were created. A brief description of each activity and tool is provided.

DD is a disaster preparedness simulation exercise. The event originated in the College of Nursing in 2007 and currently includes participants from Nursing, Medicine, Pharmacy, Emergency Medical Technicians, Physical Therapy Assistants and Radiology, as well as standardized patients and volunteers. The length of the exercise is one day with one disaster scenario conducted in the morning and repeated in the afternoon. Health professions students interact in teams to triage, deliver, and manage scenario-based patient care situations. Each health profession has discipline-specific objectives for the event but interprofessional objectives including communication and teamwork competencies were not explicitly stated until recently.

The IPE Team Observation Instrument was developed based on the IPEC competencies and was utilized to document the demonstration of the competencies throughout DD. The instrument consists of 20 items divided into four IPEC domains. Three evaluators observed the DD simulation and rated each item as demonstrated, not demonstrated, or not applicable. The evaluators had experience observing and were familiar with the instrument, so the researchers decided to utilize percent agreement instead of Cohen's Kappa for inter-rater reliability (3). The total percent agreement across all items on the observation instrument was 93%.

The IPHCE course began in 2010 and introduced health professions students to ethical issues in health care. IPHCE was a collaborative course required by the Colleges of Medicine and Nursing and was an elective for Pharmacy. The curricular activity consisted of didactic sessions and small group case discussions. Small groups were made up primarily of medical students with one or two students from other disciplines. Some of the course objectives were: analyze the impact of ethical issues on health care delivery, examine interprofessional approaches to health care and work in interprofessional groups, and apply concepts of professional practice to individual professional roles.

Since the IPHCE course no longer exists in its original format and could not be observed, a checklist was created and used to analyze the course syllabus and materials. The 42-item checklist consists of all of the IPEC competencies and sub-competencies. Two evaluators who were involved in the course and two outside evaluators reviewed the course materials and completed the checklist by placing an X in the box if the item was addressed. Inter-rater reliability and total percent agreement across all items in the checklist was 90% for the first pair of evaluators who participated in the course while the inter-rater reliability for the pair of outside evaluators who analyzed course materials but did not participate in the course was 70%. The total percent agreement was calculated separately, so that the results would not be skewed by those who had working knowledge beyond document analysis.

## Results

The DD observation instrument had a Cronbach's α reliability of 0.81 and yielded data indicating the following frequencies: Values/Ethics 68%, Roles/Responsibilities 71%, Interprofessional Communication 81%, and Team/Teamwork 74%. The IPHCE checklist had a Cronbach's α reliability of 0.82. The frequencies with which the IPEC competencies appeared to be addressed in the IPHCE course include: Values/Ethics 100%, Roles/Responsibilities 22%, Interprofessional Communication 38%, and Team/Teamwork 27%. Both DD and IPHCE facilitated the development of the IPEC competencies as indicated in (Table 1).

**Table 1:** Core competencies for interprofessional collaborative practice

Core competencies	Disaster Day	IPHCE
Values/Ethics – work with individuals of other professions to maintain a climate of mutual respect and shared values	X	X
VE1. Place the interests of patients and populations at the center of interprofessional healthcare delivery	X	X
VE2. Respect the dignity and privacy of patients while maintaining confidentiality in the delivery of team-based care	X	
VE3. Embrace the cultural diversity and individual differences that characterize patients, populations, and the healthcare team	X	X
VE4. Respect the unique cultures, values, roles/responsibilities, and expertise of other health professions	X	X
VE5. Work in cooperation with those who receive care, those who provide care, and others who contribute to or support the delivery of prevention and health services	X	X
VE6. Develop a trusting relationship with patients, families, and other team members	X	
VE7. Demonstrate high standards of ethical conduct and quality of care in one's contributions to team-based care	X	X
VE8. Manage ethical dilemmas specific to interprofessional patient/population centered care situations	X	X
VE9. Act with honesty and integrity in relationships with patients, families, and other team members	X	X
VE10. Maintain competence in one's own profession appropriate to scope of practice	X	X
Roles/responsibilities – use the knowledge of one's own role and those of other professions to appropriately assess and address the healthcare needs of the patients and populations served	X	X
RR1. Communicate one's roles and responsibilities clearly to patients, families, and other professionals	X	
RR2. Recognize one's limitations in skills, knowledge, and abilities	X	X
RR3. Engage diverse healthcare professionals who complement one's own professional expertise as well as associated resources to develop strategies to meet specific patient care needs	X	
RR4. Explain the roles and responsibilities of other care providers and how the team works together to provide care	X	
RR5. Use the full scope of knowledge, skills, and abilities of available health professionals and healthcare workers to provide care that is safe, timely, efficient, effective, and equitable	X	
RR6. Communicate with team members to clarify each member's responsibility in executing components of a treatment plan or public health intervention	X	
RR7. Forge interdependent relationships with other professions to improve care and advance learning	X	
RR8. Engage in continuous professional and interprofessional development to enhance team performance		X
RR9. Use unique and complementary abilities of all members of the team to optimize patient care	X	
Communication – communicate with patients, families, communities, and other health professionals in a responsive and responsible manner that supports a team approach to the maintenance of health and the treatment of disease	X	X
CC1. Choose effective communication tools and techniques, including information systems and communication technologies, to facilitate discussions and interactions that enhance team function		X
CC2. Organize and communicate information with patients, families, and health care team members in a form that is understandable, avoiding discipline-specific terminology when possible	X	X
CC3. Express one's knowledge and opinions to team members involved in patient care with confidence, clarity, and respect working to ensure common understanding of information and treatment and care decisions	X	X
CC4. Listen actively, and encourage ideas and opinions of other team members	X	X
CC5. Give timely, sensitive, instructive feedback to others about their performance on the team, responding respectfully as a team member to feedback from others	X	X
CC6. Use respectful language appropriate for a given difficult situation, crucial conversation, or interprofessional conflict	X	X
CC7. Recognize how one's own uniqueness, including experience level, expertise, culture, power, and hierarchy within the healthcare team contributes to effective communication, conflict resolution, and positive interprofessional working relationships	X	X
CC8. Communicate consistently the importance of teamwork in patient-centered and community focused care	X	X
Team/teamwork–apply relationship-building values and the principles of team dynamics to perform effectively in different team roles to plan and deliver patient-/population-centered care that is safe, timely, efficient, effective, and equitable	X	X
TT1. Describe the process of team development and the roles and practices of effective teams	X	
TT2. Develop consensus on the ethical principles to guide all aspects of patient care and team work	X	X
TT3. Engage other health professionals – appropriate to the specific care situation – in shared patient-centered problem-solving	X	
TT4. Integrate the knowledge and experience of other professions – appropriate to the specific care situation – to inform care decisions, while respecting patient and community values and priorities/preferences for care	X	
TT5. Apply leadership practices that support collaborative practice and team effectiveness	X	X
TT6. Engage self and others to constructively manage disagreements about values, roles, goals, and actions that arise among healthcare professionals and with patients and families	X	X
TT7. Share accountability with other professions, patients, and communities for outcomes relevant to prevention and health care	X	
TT8. Reflect on individual and team performance for individual, as well as team, performance improvement	X	
TT9. Use process improvement strategies to increase the effectiveness of interprofessional teamwork and team-based care	X	
TT10. Use available evidence to inform effective teamwork and team-based practices	X	
TT11. Perform effectively on teams and in different team roles in a variety of settings.	X	X

## Discussion and conclusions

The observation instrument and the checklist both appear to be acceptable tools for determining competency alignment. Based on our analysis, DD appears to meet more of the IPEC competencies than the IPHCE course. However, the activities were evaluated using different tools, which is a limitation. Due to this, we decided to conduct a *post hoc* DD analysis using the IPHCE checklist. The checklist data suggest that the IPHCE course may have addressed and contributed to Values/Ethics, Roles/Responsibilities, Communication, and Team/Teamwork to some degree, but DD appears to meet more of the competencies in the four domains.

Even though DD mapped to more of the IPE competencies, having IPHCE and other IPE activities available is ideal since learning in a variety of interprofessional settings is optimal (4). Utilizing the checklist and observation tool that we developed will help to ensure that all competencies are addressed. Future research may investigate whether competency alignment using these tools contributes to sustainability.
